# Subclinical and clinical hypothyroidism and non-alcoholic fatty liver disease: a cross-sectional study of a random population sample aged 18 to 65 years

**DOI:** 10.1186/s12902-015-0030-5

**Published:** 2015-08-15

**Authors:** Ulla Ludwig, Daniela Holzner, Christian Denzer, Artur Greinert, Mark Martin Haenle, Suemeyra Oeztuerk, Wolfgang Koenig, Bernhard Otto Boehm, Richard Andrew Mason, Wolfgang Kratzer, Tilmann Graeter, the EMIL-Study

**Affiliations:** Department of Internal Medicine I, Center for Internal Medicine, University Medical Center Ulm, Albert-Einstein-Allee 23, 89081 Ulm, Germany; Department of Pediatrics and Adolescent Medicine, University Medical Center Ulm, Eythstr. 24, 89075 Ulm, Germany; Department of Internal Medicine II, Center for Internal Medicine, University Medical Center Ulm, Albert-Einstein-Allee 23, 89081 Ulm, Germany; Deutsches Herzzentrum München, Technische Universität München, Munich, Germany; DZHK (German Centre for Cardiovascular Research), partner site Munich Heart Alliance, Munich, Germany; Lee Kong Chian School of Medicine, Nanyang Technological University, Singapore, Singapore; Imperial College London, London, UK; Louis Stokes Cleveland Department of Veterans Affairs Medical Center, 10701 East Boulevard, Cleveland, OH 44106 USA; Department of Diagnostic and Interventional Radiology, University Medical Center Ulm, Albert-Einstein-Allee 23, 89081 Ulm, Germany

**Keywords:** NAFLD, Thyroid, Hypothyroidism, NASH, Cross-sectional studies

## Abstract

**Background:**

Non-alcoholic fatty liver disease (NAFLD) is one of the most common disorders of the liver worldwide. Recently, a correlation between thyroid dysfunction and NAFLD has been discussed. Objective of the present study was to investigate the association between thyroid dysfunction and hepatic steatosis.

**Methods:**

Data from 2,445 subjects (51.7 % females) aged 18 to 65 years participating in a population-based cross-sectional study were assessed based on a standardized questionnaire and documentation of physical, biochemical and ultrasonographic findings. After application of exclusion criteria, a total of 1,276 subjects were included in the study collective. The influence of potential factors on the development of hepatic steatosis was assessed using multivariate logistic regression.

**Results:**

The prevalence of hepatic steatosis in the study collective was 27.4 % (n = 349). The serum thyroxin (TT4) concentration in subjects with hepatic steatosis was reduced (p = 0.0004). Adjusting for age, or BMI, there was an increased prevalence of hepatic steatosis in subjects with reduced TT4 concentrations (p = 0.0143; p = <.0001).

**Conclusions:**

The findings of the present study confirm an association between both subclinical and clinical hypothyroidism and hepatic steatosis

## Background

Non-alcoholic fatty liver disease (NAFLD) represents one of the most common chronic disorders of the liver in the Western industrialized nations [1–4]. Its prevalence worldwide is estimated at 20 % -30 % [5–7]. NAFLD subsumes a variety of entities ranging from simple fatty liver or hepatic steatosis, to non-alcoholic steatohepatitis (NASH) and cirrhosis of the liver [8–10] and is associated with the risk of malignant degeneration to hepatocellular carcinoma (HCC) and the increased necessity of liver transplantation [11, 12]. A central role in the development of NAFLD has been ascribed to the metabolic syndrome, whose main characteristics, such as obesity, insulin resistance and/or type-2 diabetes mellitus and dyslipidemia are closely associated to NAFLD [8]. Not surprisingly, there is also an association between NAFLD and cardiovascular disorders [1, 2].

Increasingly, a correlation between thyroid dysfunction, especially clinical or subclinical hypothyroidism, and NAFLD has been discussed [6, 13–16]. Hormones synthesized in the thyroid gland play an important role in the regulation of diverse metabolic processes. Disturbances in thyroid hormone concentrations may promote hyperlipidemia and obesity, thus contributing to NAFLD [13, 17]. Early identification of at-risk patients is important since treatment of the hypothyroidism may reduce the risk of NAFLD and potential complications [18]. It was the objective of the present study to investigate the association between thyroid dysfunction and hepatic steatosis in an epidemiological cross-sectional study in a random population-based sample of subjects aged 18 to 65 years.

## Methods

### Study population

**Fig. 1:**
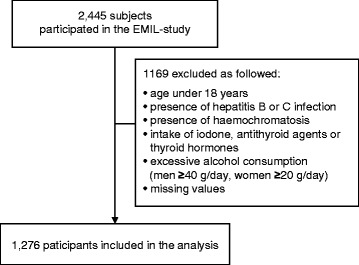
Flow of the subjects across the study. The base collective for the present study consisted of the 2,445 subjects of the EMIL study. Of these, 258 subjects were excluded due to age < 18 years; 69 due to excessive alcohol consumption (>40 g/day in males and > 20 g/day in females); 146 due to past or current hepatitis B or hepatitis C infections;intake of iodone (n = 344), antithyroid agents (n = 2) or thyroid hormones (n = 437); and 1 due to hemochromatosis. Incomplete data, laboratory values or other data were also exclusion criteria. Each box represents an exclusion criterion and contains the corresponding number of subjects in relation to the total collective. An individual subject may meet multiple exclusion criteria. For the present study, the resulting collective consisted of 1,276 individuals

A cross-sectional survey assessing the prevalence of ***E****chinococcus****M****ultilocularis****I****nfection and other medical disorders in****L****eutkirch* (EMIL-Study), was conducted in Leutkirch, Germany in 2002. Initially, 4,000 of the total 12,475 residents were randomly selected by the staff of the municipal registry office from the roster of inhabitants. Out of these 4,000 persons, 107 were excluded because their address was unknown or they had not given their informed consent. A total of 2,445 individuals finally participated in the study, corresponding to a participation rate of 62.8 % [19]. Following exclusion of subjects less than 18 years (n = 258) and subjects with incomplete laboratory results (n = 230), those with past or present hepatitis B or hepatitis C virus infections (n = 146), hemochromatosis (n = 1) or elevated alcohol consumption (>40 g/day in males and > 20 g/day in females; n = 69), intake of iodone (n = 344), antithyroid agents (n = 2) or thyroid hormones (n = 437), missing ultrasonographic data on hepatic steatosis (n = 16), missing data on BMI (n = 11) or metabolic syndrome (n = 77), a total of 1,276 subjects were finally included in the present analysis (Fig. 1). This study collective was then divided, based on the diagnosis of NAFLD, into an NAFLD group (n = 349) and a healthy control group (n = 927).

The study was conducted in accordance with the principles of good clinical practice and the Declaration of Helsinki. It was approved by the ethics committee of the Medical Association of Baden-Württemberg. All participants provided written informed consent.

### Examination methods and anthropometric data

Patient history, including demographics, leisure activities, past medical history, family medical history, medication history as well as nicotine and alcohol use and nutritional habits were compiled and documented using a standardized interview. Body height, body weight, hip, and waist circumference were measured. The body-mass index (BMI) and the waist-to-hip ratio (WHR) were calculated according to recommendations of the World Health Organization (WHO).

### Laboratory testing

About 25 ml of whole blood was obtained from each study participant through phlebotomy of the cubital vein. Random glucose, alanine aminotransferase (ALT), aspartate aminotransferase (AST), γ-glutamyl transferase (GGT), alkaline phosphatase (AP), C-reactive protein (CRP), albumin, cholesterol, high density lipoprotein (HDL), transferrin and ferritin were measured using the Dimension XL unit (Dade Behring Inc., Newark, DE 19714, USA). Fibrinogen and coeruloplasmin were measured using the Siemens Dade Behring BII Nephelometer (Siemens Healthcare Diagnostics GmbH; Ludwig-Erhard-Straße 12; 65760 Eschborn, Germany). The concentration of low density lipoprotein (LDL) was calculated using the Friedewald formula: LDL cholesterol = total cholesterol–HDL–(triglycerides*0.45). Thyroid-stimulating hormone (TSH)**,** triiodothyronine (TT3), thyroxine (TT4) and anti-thyroid autoantibodies (Anti-TPO) were measured using an Elecsys® 2010 Disk & Rack Analyzers (Roche Diagnostics, 9115 Hague Road, Indianapolis, USA). Each parameter was measured twice. All assays were performed according to the manufacturer’s recommendations. Regular precision controls were performed to assure proper functioning of all laboratory equipment.

### Criteria for thyroid dysfunction and metabolic syndrome

The diagnosis of *subclinical* hypothyroidism was made in subjects with TSH concentrations ≥ 34 IU/ml and normal thyroid hormone concentrations (TT4: 12.8-20.4 pmol/l; TT3: 3.92-6.74 pmol/l). The diagnosis of clinically manifest hypothyroidism required reduced TT4 concentrations (<12.8 pmol/) and elevated TSH levels (TSH ≥ 34 IU/ml).

Metabolic syndrome was defined according to modified U.S. National Cholesterol Education Program Adult Treatment Panel III criteria (ATP III), whereby at least three of the following criteria had to be met: waist circumference >102 cm in males or >88 cm females; serum triglyceride (TG) concentration ≥ 1.7 mmol/l; serum HDL cholesterol ≤ 1.28 mmol/l (males, 1.0 mmol/l; females, 1.3 mmol/l); history of hypertension and random blood glucose ≥ 160 mg/dl (fasting glucose ≥ 110 mg/dl) or confirmed diabetes mellitus [20].

### Ultrasound examination and criteria for hepatic steatosis

The ultrasound examinations were performed under standardized conditions by specially trained examiners using four identical HDI 5000 diagnostic ultrasound units (ALTATL Ultrasound, Philips Medical Systems, P.O. Box 3003, Bothell, WA, USA). Unclear or pathological findings were reviewed by an experienced supervisor (>4,000 ultrasonographic examinations per year). As far as possible, identical settings were maintained for all units. Findings were documented using a standardized recording questionnaire. Examinations included assessment of the liver, gallbladder, kidneys and spleen. The liver was assessed with respect to size, the presence of focal lesions, and the presence of hepatic steatosis. The diagnosis of hepatic steatosis was made based on the basis of criteria established by Saverymuttu *et al.* [21], Hamaguchi *et al.* [22] and Charatcharoenwitthaya *et al.* [23]. The hepatic parenchyma was compared with the renal parenchyma under consideration of the dorsal echo attenuation, visualization of the diaphragm and ability to assess the intrahepatic vessels. The degree of steatosis was assigned to classes of “none” (grade 0), “mild” (grade I), “moderate” (grade II) and “severe” (grade III).

### Statistical analysis

Statistical calculations were performed using the SAS 9.2 statistics software package (SAS Institute Inc., Cary, North Carolina, USA). Data were first analyzed descriptively. Mean and standard deviation were determined for continuous variables. Categorical data were presented with absolute and relative frequencies. In order to detect differences between subjects with and without hepatic steatosis, the Wilcoxon rank-sum test was used for continuous variables, while, for categorical variables, the *χ*^2^ test or, when the number of cases was too small, Fisher’s exact test were used. In order to identify possible correlations between thyroid hormone levels and demographic, anthropometric and biochemical parameters, we calculated the Spearman rank correlation. In a further analytic step, subjects were divided into four equal groups (quartiles) on the basis of thyroid hormone concentrations (TSH, TT4, TT3; Table 1). The Kruskal-Wallis test was used to identify differences between the four groups. Bivariate and multivariate logistic regression served to identify correlations with hepatic steatosis and other potential risk factors. All tests were two-tailed. A *P* value of less than 0.05 was considered statistically significant.

**Table 1:** Breakdown of thyroid hormone parameters in quartiles in the present study

	Quartile 1	Quartile 2	Quartile 3	Quartile 4
TSH	<1.0	1.01-1.49	1.50-2.18	>2.19
TT4	<74.5	74.6-83.9	84.0-94.2	>94.3
TT3	<1.40	1.41-1.57	1.58-1.81	>1.82

## Results

### Hepatic steatosis

The study collective included 1,276 subjects (47.2 %, females; 52.8 %, males), whose mean age stood at 40.7 ± 12.7 years (40.0 ± 12.7 years, women; 41.3 ± 12.6 years, men). The characteristics of the study population are presented in Table 2. A diagnosis of hepatic steatosis was made in 349 subjects. The prevalence of ultrasonographically diagnosed hepatic steatosis was significantly higher in males (70.8 %; n = 247) than in females (29.2 %; n = 102; Table 2). The NAFLD group was characterized by a higher BMI, WHR, arterial hypertension and higher prevalence of metabolic syndrome according to ATPIII criteria (p < 0.001). The biochemical parameters ALT, AST, GGT were also significantly higher in the group with hepatic steatosis (Table 2).

**Table 2:** Characteristics of study subjects with and without NAFLD

Variables	Subjects with NAFLD (n = 349) Mean ± SD	Subjects without NAFLD (n = 927) Mean ± SD	*P* value
Gender, *n (%)*			
*female*	102 (29.2)	500 (53.7)	<.0001
*male*	247 (70.8)	427 (46.1)	
Age	47.7 ± 11.5	38.0 ± 12.1	<.0001
BMI	29.7 ± 4.7	24.0 ± 3.7	<.0001
WHR	0.9 ± 0.1	0.8 ± 0.1	<.0001
ALT	20.9 ± 10.3	13.5 ± 5.8	<.0001
AST	11.2 ± 5.1	9.0 ± 2.6	<.0001
GGT	20.5 ± 20.6	11.0 ± 10.5	<.0001
TSH (μU/ml)	1.8 ± 1.4	1.8 ± 3.5	0.6381
TT3 (nmol/l)	1.6 ± 0.3	1.6 ± 0.3	0.3293
TT4 (nmol/l)	83.2 ± 15.6	92.0 ± 17.4	0.0004
Anti-TPO (IU/ml)	23.9 ± 72.1	23.9 ± 74.0	0.4063
Diabetes, n (%)	22 (6.3)	8 (0.9)	<.0001
Metabolic syndrome, n (%)	65 (18.6)	15 (1.6)	<.0001
Hypertension, n (%)	99 (28.4)	67 (7.2)	<.0001

*BMI = body-mass-index; WHR = waist to hip ratio; ALT = Alanine transaminase; AST = Aspartate transaminase; GGT = Gamma-glutamyl transferase; TSH = thyroid-stimulating hormone; TT3 = triiodothyronine; TT4 = thyroxine; Anti-TPO = anti-thyroid autoantibodies*

### Association of NAFLD with thyroid hormone concentrations

Hypothyroidism was diagnosed in 4.1 % (n = 52) subjects. Of these, 34 subjects met criteria for subclinical, 18 for clinically manifest hypothyroidism. In the subgroup with hepatic steatosis, 1.3 % of subjects (n = 16; 7 females, 9 males) exhibited thyroid hormone levels consistent with hypothyroidism. Of these, eight subjects (0.6 %) met criteria for subclinical, eight (0.6 %) for clinically manifest hypothyroidism (Table 2). Among subjects with both hepatic steatosis *and* elevated serum transaminases (n = 125), six (4.8 %) exhibited thyroid hormone levels consistent with hypothyroidism. Of these, five subjects met criteria for subclinical, one for clinically manifest hypothyroidism. By comparison, in the control group, 46 subjects (25 females, 21 males) exhibited reduced thyroid hormone concentrations. Of these, 29 subjects met criteria for subclinical, 17 for clinically manifest hypothyroidism. With respect to the thyroid hormone concentrations, there was a significant difference only for TT4 levels (p = 0.0.0004; Table 2). The mean values for TSH, TT3 and Anti-TPO did not differ significantly between the groups.

**Fig. 2:**
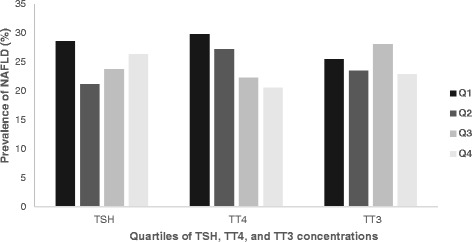
Prevalence of non-alcoholic fatty liver disease (NAFLD) in relation to thyroid function in the present study. The figure plots the thyroid hormone concentrations in their respective quartiles (*x*-axis) against NAFLD prevalence rates in percent (*y*-axis). NAFLD prevalence rates show a downward trend with increasing TT4 concentrations. In addition, a positive trend is also seen for NAFLD prevalence rates with increasing TSH levels in the first quartile

In order to assess the prevalence of NAFLD, the findings for TSH, TT3 and TT4 were broken down into quartiles (Table 1) and the individual subjects analyzed. Fig. 2 displays a downward trend in the prevalence rate of hepatic steatosis with increasing TT4 values. By contrast, except in the first quartile, the prevalence rate for hepatic steatosis showed an upward trend with increasing TSH concentrations.

### Association of NAFLD with other variables

Males had a 2.8-fold increased risk of NAFLD compared with females (OR = 2.836; CI = 2.177-3.694). There was a significant correlation between NAFLD and age (p < 0.0001). Furthermore, NAFLD shows a significant association with BMI (p < 0.0001) and WHR (p < 0.0001). There was also a significant correlation between the presence of NAFLD and both metabolic syndrome (OR = 13.913; 95 % -CI 7.813 24.773; p = 0.0001) and diabetes mellitus (OR = 7.727; 95 % -CI = 3.407-17.525; p < 0.0001).

### Association of NAFLD with thyroid hormones

In the univariate analysis, TSH and TT3 show no significant association with hepatic steatosis (p = 0.7453; p = 0.1142). For TT4 a significant correlation with NAFLD was shown (p = 0.008; OR = 0.987; 95 % -CI = 0.979-0.995). After adjusting for age or BMI, a significant association could be shown for TT4 and hepatic steatosis. Adjusted for WHR or gender, no significant association could detected (Table 3).

**Table 3:** Association of hepatic steatosis with thyroid hormone

	OR (95 % -CI)	p-value
*bivariate*		
TSH	0.992 (0.945-1.042)	0.7453
TT3	0.738 (0.506-1.076)	0.1142
TT4	0.987 (0.979-0.995)	0.0008
*multivariate*		
TT4 *adjusted for age*	0.990 (0.982-0.998)	0.0143
TT4 *adjusted for BMI*	0.988 (0.979-0.997)	<.0001
TT4 *adjusted for WHR*	0.997 (0.987-1.006)	0.5048
TT4 Adjusted for gender	0.994 (0.985-1.002)	0.1172
TT4 * Adjusted for BMI, age, WHR, gender*	0.994 (0.984-1.005)	0.2959

*TSH = thyroid-stimulating hormone; TT3 = triiodothyronine; TT4 = thyroxine; BMI = body-mass-index; WHR = waist to hip ratio*

## Discussion

Over the past decade, beginning with a study by Liangpunsakul and Chalasani in 2003 [14], the association between thyroid dysfunction and non-alcoholic fatty liver disease (NAFLD) has increasingly become a focus of research. After some controversial reports [24, 25], numerous studies have confirmed an association between thyroid function and NAFLD [13, 15, 26, 27]. Unfortunately, most studies have been characterized by either a relatively small or selected study collective, or by a gender imbalance [6, 14, 15, 28–30]. In addition, study subjects were in many cases patients rather than being a collective representative of the general population [14, 29, 30]. By contrast, the present study, like those of Chung *et al.*, Ittermann *et al.* und Zhang *et al.*, is characterized by a balanced gender distribution and its large study collective representative of the general population [13, 27, 31]. In addition, with a mean age of 40.7 ± 12.7 years, our collective is one of the younger populations studied, and includes the age group with the highest prevalence of NAFLD [5, 6].

The findings of the present study agree in general with those reported from the cross-sectional study of Ittermann *et al.* In both studies, a significant inverse association between the FT4 concentration of NAFLD could be demonstrated, while no significant association could be identified for TT3 or TSH [31]. This underscores the importance of the TT4 or FT4 concentration as a marker for hepatic steatosis in the general population. By contrast, the TT3 concentration, both in the present study and in those of Ittermann *et al.* and Xu *et al.*, had no identified value as a marker [6, 31]. This finding could related to an inhibition of the conversion of TT4 to TT3, possibly explaining the subordinate diagnostic role of the TT3 or FT3. Even the study of Chung *et al.*, which presented clear evidence of the association between hypothyroidism and NAFLD, did not ascribe any diagnostic value to the TT3 concentration [13].

Beside the inverse association with FT4, Chung *et al.* and Xu *et al.* identified a positive association between NAFLD and TSH [6, 13]. This association was observed in other studies [28, 29]. The difference between these studies and the present investigation may be the result of a divergent classification of study subjects regarding thyroid function or differences in recruitment of subjects from population-based sources or from a patient collective. Data reported by Carulli *et al.* and Pagadala *et al.* suggest, in addition, that the TSH concentration is associated with the severity of the hepatic steatosis [15, 28].

The above observations point to a possible correlation between thyroid dysfunction and NAFLD. This correlation may be explained, on the one hand, by a significant association between thyroid dysfunction, a hypothyroid metabolic state and the metabolic syndrome [28, 32, 33]. Metabolic syndrome, in turn, is associated with NAFLD [1, 7, 9] and would point indirectly to a possible correlation between thyroid dysfunction and NAFLD. On the other hand, the correlation may be grounded in the association of reduced TT4 levels with hypertriglyceridemia and overweight. Beside studies which confirm the association between thyroid dysfunction, especially the hypothyroid metabolic state, and metabolic syndrome, there are studies with controversial findings [34, 35]. These studies highlight the correlation between NAFLD and thyroid dysfunction. For example, the study by Roos *et al.* shows a correlation between the FT4 level and triglycerides [33]. Our study would confirm this finding, as our data show a significant association between the TT4 level and triglycerides (p = 0.0027). These observations agree with the findings of other studies that suggest a correlation between hypothyroidism and hyperlipidemia [33, 36]. The increase in triglycerides in patients with hypothyroidism is explained by the reduced hepatic activity of triglyceride lipase [32, 33] and increased fatty acid oxidation. Loria *et al.* come to the conclusion that hepatic steatosis may develop from hypothyroidism-induced hyperlipidemia and overweight [17]. The development of hepatic steatosis may be explained by an increase influx of triglycerides and an imbalance between the in- and outflow of other lipids in the liver.

Because thyroid dysfunction, especially the hypothyroid metabolic state, affects the overall metabolism and may contribute to the development of hepatic steatosis and more serious forms of NAFLD, future interventional studies, similar to that of Ineck *et al.* should focus on treatment of thyroid dysfunction [18].

The present study has some limitations. Because of the choice of study design, it was possible to investigate associations but not causalities. While liver biopsy represents the gold standard for diagnosis of NAFLD [1], the diagnosis of hepatic steatosis in our study was made using ultrasonography. Also, because serum insulin levels were not determined in fasting subjects and thus could not be used, insulin resistance was not assessed in this study as a possible factor impacting the association between thyroid dysfunction and NAFLD.

## Conclusions

In summary, the results of the present study agree in many points with the findings of numerous publications and confirm a correlation between low TT4 concentrations and hepatic steatosis in a study collective representative of the general population. The prevalence of hepatic steatosis rises significantly with reductions in TT4 concentrations. A low TT4 concentration appears to be an independent risk factor for hepatic steatosis. Future studies should further elucidate the impact of thyroid hormone parameters on NAFLD. This could serve to clarify the underlying pathogenetic mechanisms and provide potentially important data for therapeutic and preventive measures.

## Abbreviations

ALT: Alanine Aminotransferase
Anti-TPO: Anti-Thyroid Peroxidase antibodies
AST: Aspartate Aminotransferase
ATP III: Adult Treatment Panel III
BMI: Body-Mass Index
FT3: Free Triiodthyronin
FT4: Free Thyroxin
GGT: γ-Glutamyl transferase
NAFLD: Non-Alcoholic Fatty Liver Disease
NASH: Non-Alcoholic Steatohepatitis
nmol: Nanomol
SD: Standard Deviation
TT3: Triiodthyronin
TT4: Thyroxin
TSH: Thyroid-Stimulating Hormone
WHO: World Health Organization
WHR: Waist-to-Hip-Ratio
